# Celebrating 10 Years of AORTA

**DOI:** 10.1055/s-0042-1745865

**Published:** 2022-05-31

**Authors:** John A. Elefteriades, Bulat A. Ziganshin

**Affiliations:** 1 AORTA Journal Editorial Office, Aortic Institute at Yale New Haven Hospital, Yale University School of Medicine, New Haven, Connecticut

We are pleased to report that AORTA has now reached its 10-year anniversary.

We wish to express appreciation to many individuals.

**Fig. 1:**
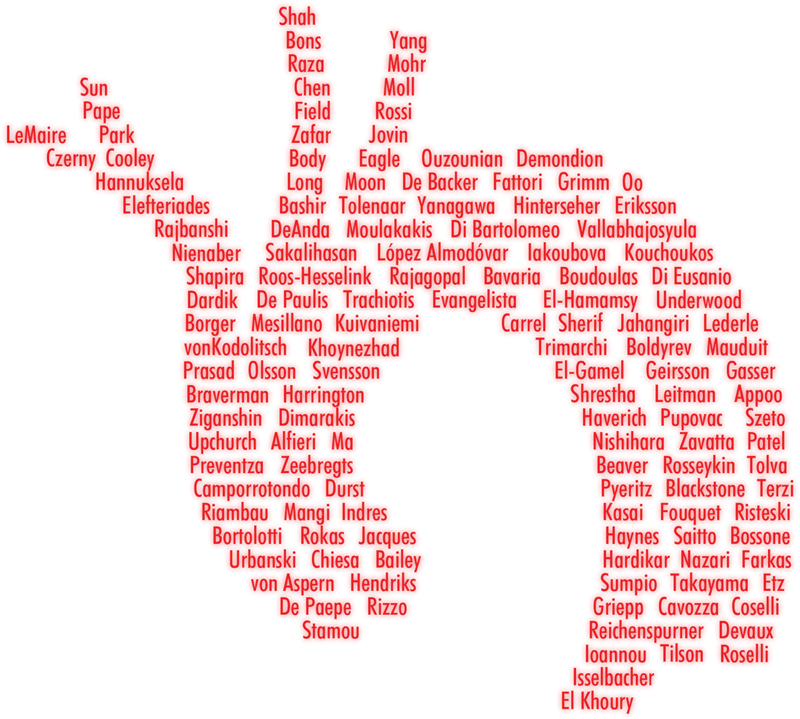
Artistic rendition of the partial list of authors who have published in *AORTA* .

We wish to thank especially our authors, who have submitted interesting and meaningful manuscripts for publication. These have reflected insight, imagination, and, very often, surgical courage. The accompanying collage ( Fig. 1 ) represents just a fraction of the distinguished worldwide authorities who have published their work in *AORTA* . In 2021, we received manuscript submissions from 16 different countries (United States, United Kingdom, Turkey, Sweden, South Africa, Singapore, The Netherlands, Korea, Japan, Italy, Iran, India, Greece, Germany, Belgium, and Australia).

We express appreciation to the Editorial Board for their varied and valued efforts.

We express appreciation to our Reviewers, who have contributed immensely to enhancing submitted manuscripts toward increased scientific rigor and clarity.

We thank Dr. Steven Korn and Science International Corporation for getting us started and leading us on the proper path. Our thanks go out to our current Publishers, the Thieme Group, with special appreciation for Graham Brumfield, Senior Executive Editor at Thieme Group for his leadership, and to Jessica Sieger, Editor and Project Manager for Thieme Group, who actually make things run.

**Fig. 2:**
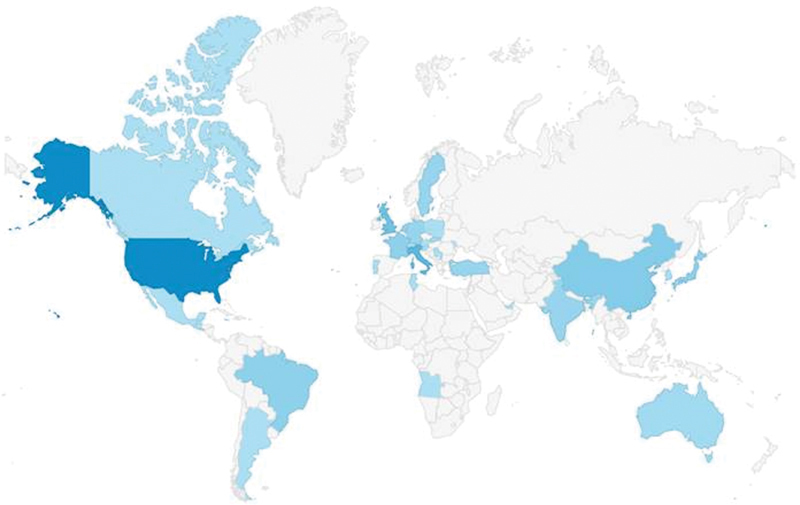
Geographic representation of the readership of *AORTA* in 2021. Readers truly spanned the globe: United States., Italy, United Kingdom, China, Brazil, France, Japan, Germany, India, The Netherlands, Sweden, Turkey, Australia, Slovakia, United Arab Emirates, Angola, Argentina, Austria, Canada, Switzerland, Guatemala, Jamaica, South Korea, Moldova, Mexico, Poland, Portugal, Serbia, Singapore, Slovenia, El Salvador, and Tunisia.

And, a special “thank you” goes out to our readers, who, as seen in the accompanying map ( Fig. 2 ), literally span the globe. In 2020, *AORTA* had 12,652 full-text reads on tieme-connect.com.

We would like to thank our sponsor Medtronic for supporting the Journal in 2021.

We would like to thank Yale New Haven Hospital for generous and enduring support.

Thank you all. We look forward to the next 10 years studying this amazing organ: the human aorta, which is “much more than a tube.” 1
